# Supplementary material for: Synergetic binary organocatalyzed ring opening polymerization for the precision synthesis of polysiloxanes
Source: Commun Chem. 2024 Mar 21;7:61. doi: 10.1038/s42004-024-01140-3 (PMC10957864; doi:10.1038/s42004-024-01140-3)
Supplement: Supplementary file 2 — Supplementary Information [file 42004_2024_1140_MOESM2_ESM.pdf]

Supplementary Methods

NMR Measurements

^{1}H NMR spectra were recorded on a Bruker AVANCE III spectrometer operating at 500 MHz. $CDCl_3$ was used as the solvent, and chemical shifts were reported relative to solvent residual signals.

SEC Measurements

SEC measurements were performed using a Waters e-2695 high-speed liquid chromatograph equipped with RI and UV detectors. THF was used as the eluent at a flow rate of 0.50 mL min^{-1} at 40 °C and a Shodex KF-603 column was employed. The calibration curve was obtained with TSK standard polystyrenes (Tosoh Co.); the M_w(LS)s were 189000, 37200, 9490, 2500, and 589.

FT-IR Measurements

Attenuated total reflectance (ATR) FT-IR spectra were recorded on a Bruker Alpha II FT-IR spectrometer equipped with an ATR module.

Rheological analyses

Rheological analyses were performed on an Anton Paar MCR 102 rheometer equipped with a Peltier temperature control device. Time-dependent analyses of G' and G'' for the PDMS samples with a thickness of 0.3 mm were conducted using a parallel plate with a diameter of 12 mm at a frequency of 5 Hz at 25 °C.

Tensile tests

Tensile tests were conducted on a Shimadzu AGS-X mechanical testing machine with a

5 N load cell operating at a crosshead speed of 100 mm min^{-1} at room temperature. The tensile modulus (E), elongation at break (ε_B), tensile strength (σ_T), and fracture energy (Γ) of a specimen were calculated with TRAPEZIUM X software built into the instrument.

Supplementary Figures

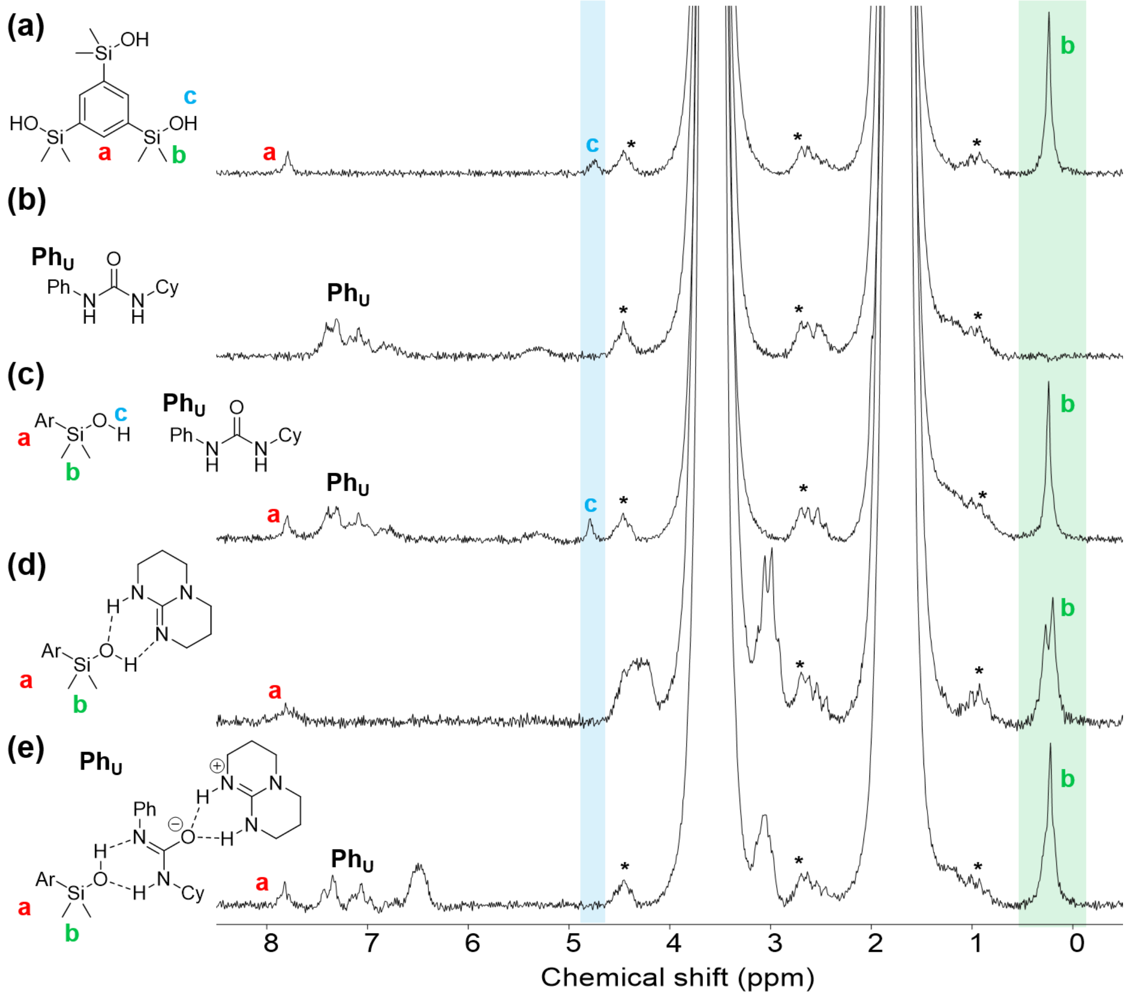

Supplementary Figure 1. ^{1}H NMR spectra (80 MHz, THF) of (a) **I₃**, (b) U(Cy), (c) **I₃** + U(Cy) ([**I₃**] : [U(Cy)] = 1 : 3), (d) **I₃** + TBD ([**I₃**] : [TBD] = 1 : 0.75), and (e) **I₃** + TBD + U(Cy) ([**I₃**] : [TBD] : [U(Cy)] = 1 : 0.75 : 3). [**I₃**] = 0.018 M. The asterisks indicate satellite signals of two THF-derived signals appearing at 1.79 and 3.62 ppm.

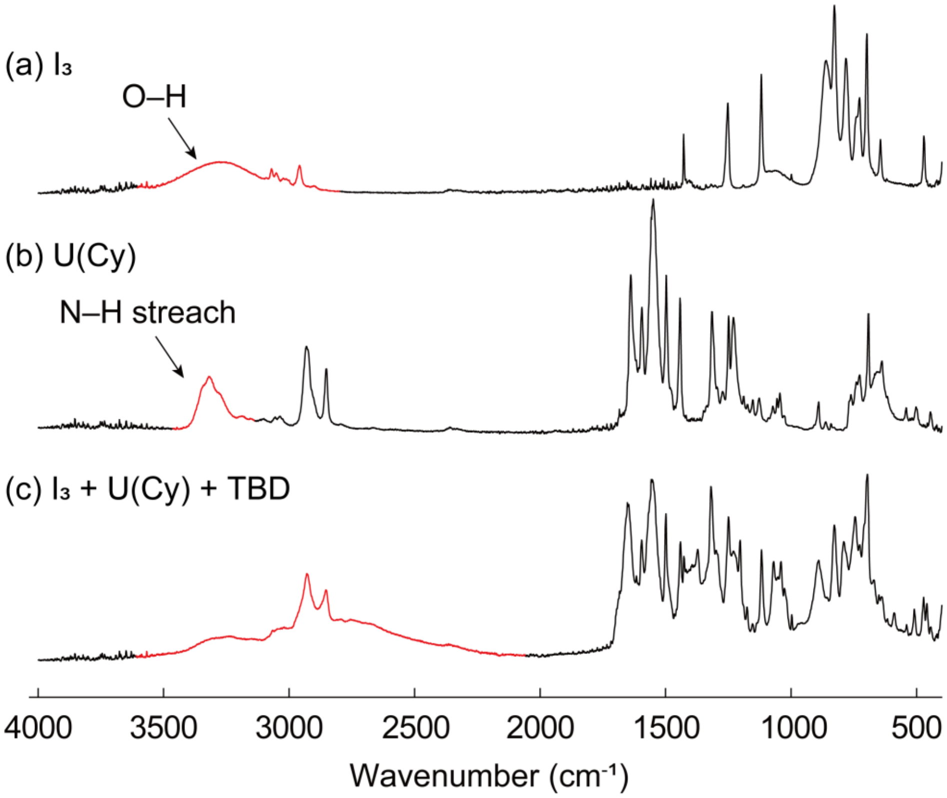

Supplementary Figure 2. IR spectra of (a) $\mathbf{I_3}$, (b) U(Cy), and (c) $\mathbf{I_3}$ + U(Cy) + TBD ($\mathbf{I_3}$: U(Cy) : TBD = 1 : 3 : 3).

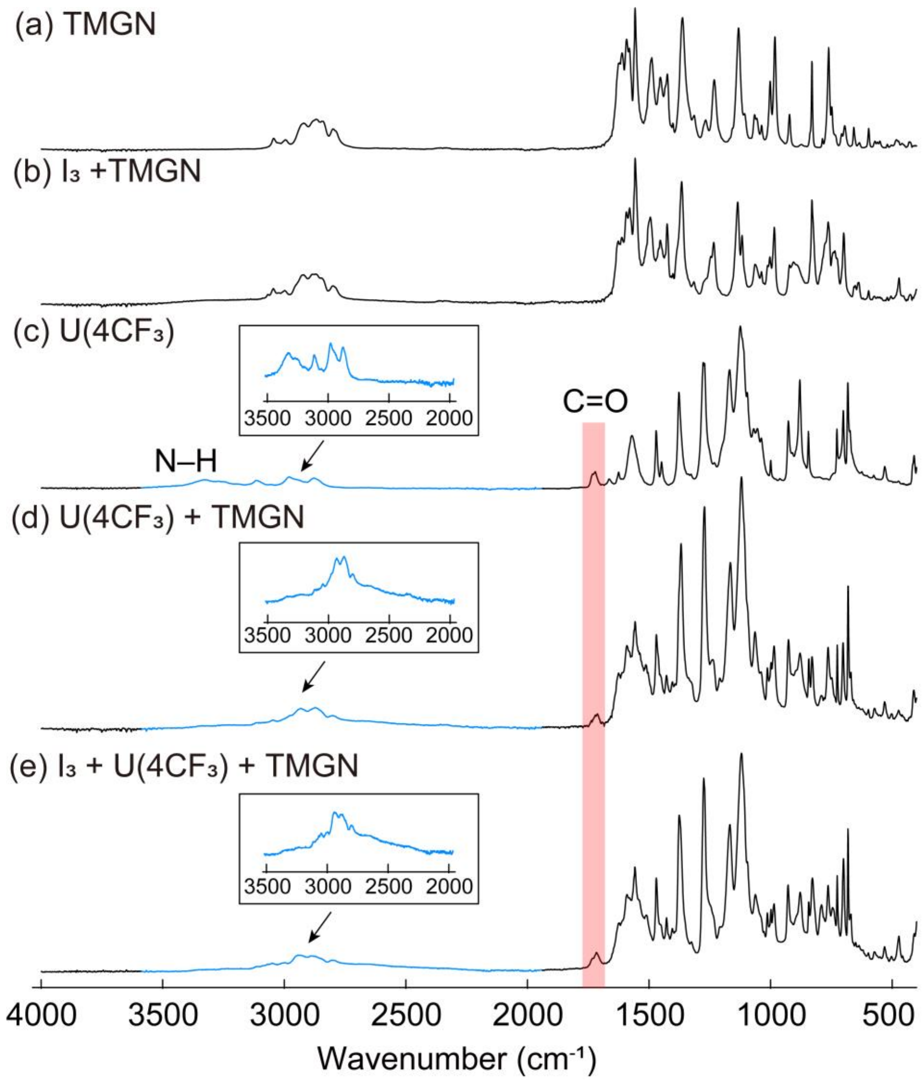

Supplementary Figure 3. IR spectra of (a) TMGN, (b) $\mathbf{I_3}$ + TMGN ($\mathbf{I_3}$: TMGN = 1 : 1), (c) U($4CF_3$), (d) U($4CF_3$) + TMGN (U($4CF_3$) : TMGN = 1 : 1), and (e) $\mathbf{I_3}$ + U($4CF_3$) + TMGN ($\mathbf{I_3}$: U($4CF_3$) : TMGN = 1 : 3 : 3).

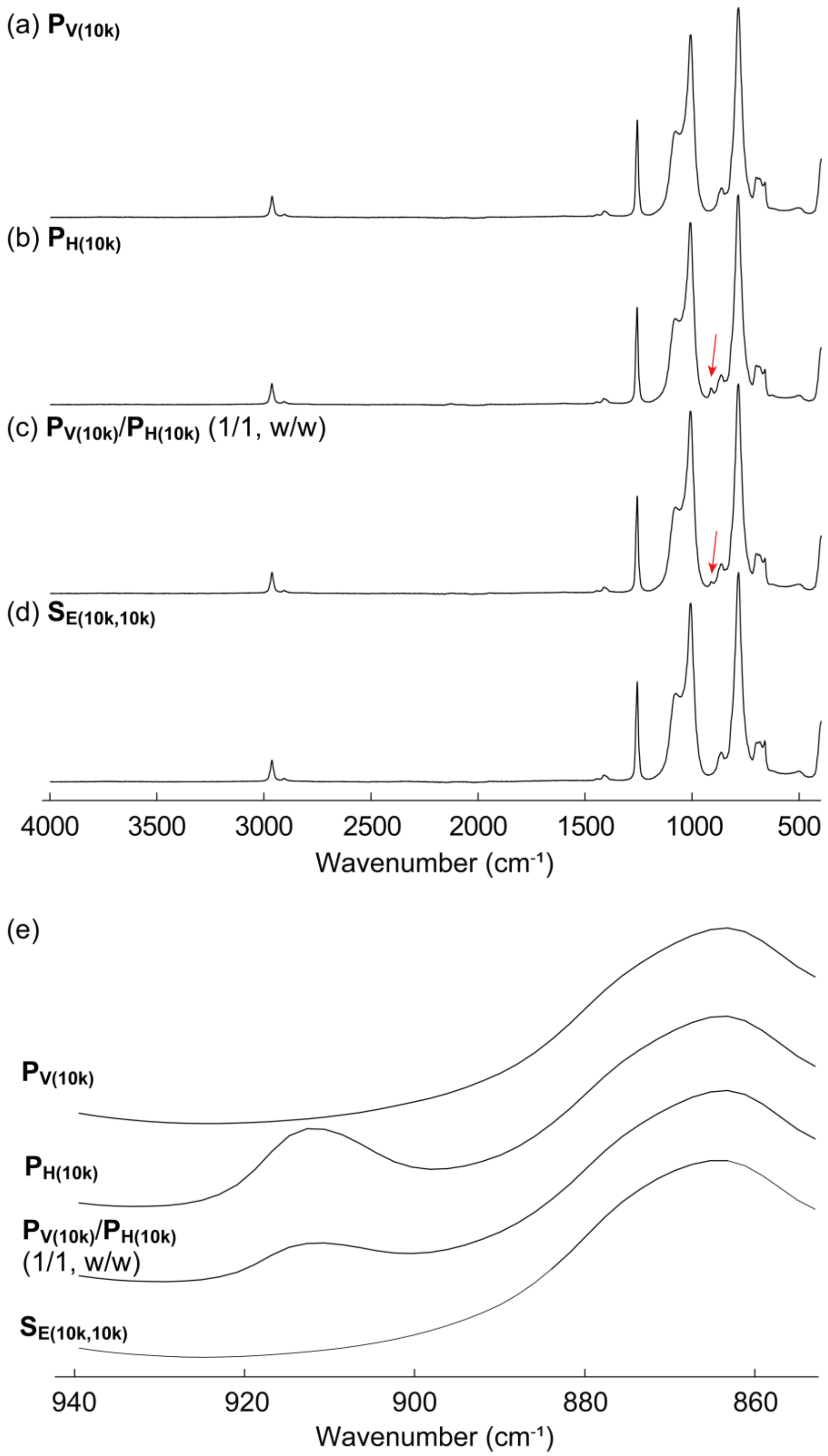

Supplementary Figure 4. IR spectra of (a) $\mathbf{P_{V(10k)}}$, (b) $\mathbf{P_{H(10k)}}$, (c) $\mathbf{P_{V(10k)}}$/$\mathbf{P_{H(10k)}}$ (1/1, w/w), (d) $\mathbf{S_{E(10k,10k)}}$. Red arrows are the indication of Si–H bending vibration at 912 cm^{-1}. (e) Magnified IR spectra of $\mathbf{P_{V(10k)}}$, $\mathbf{P_{H(10k)}}$, $\mathbf{P_{V(10k)}}$/$\mathbf{P_{H(10k)}}$ (1/1, w/w), and $\mathbf{S_{E(10k,10k)}}$. at the wavenumber range of 850–940 cm^{-1}.

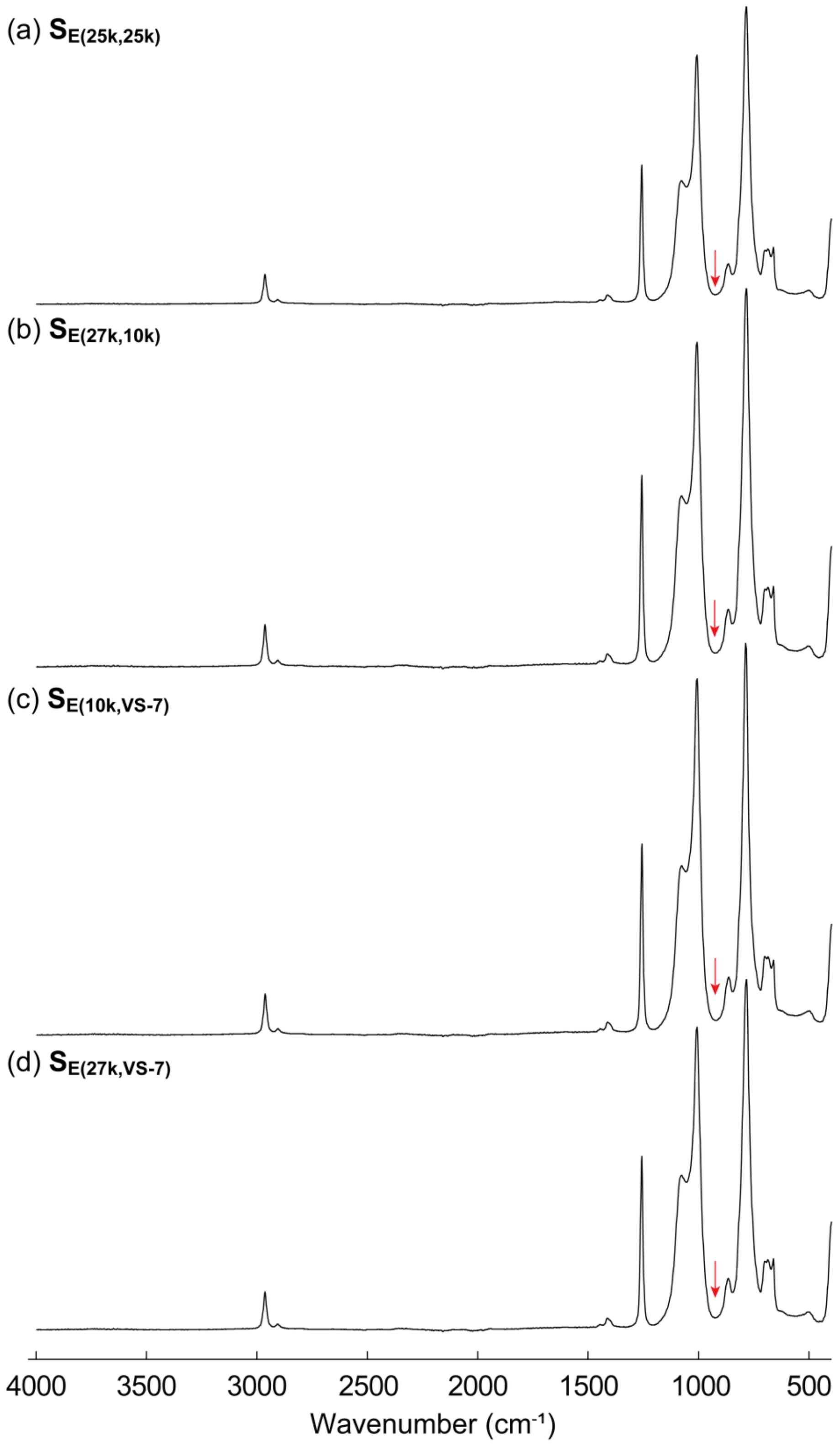

Supplementary Figure 5. IR spectra of (a) $\mathbf{S}_{\mathbf{E(25k,25k)}}$, (b) $\mathbf{S}_{\mathbf{E(27k,10k)}}$, (c) $\mathbf{S}_{\mathbf{E(10k,VS\text{-}7)}}$, and (d) $\mathbf{S}_{\mathbf{E(27k,VS\text{-}7)}}$. Red arrows indicate the disappearance of the Si–H bending vibration at 912 cm^{-1}.

Supplementary Tables

Supplementary Table 1. SEC characterization of star-shaped PDMSs.

Code	Polymer	M_n[a]	M_w[b]	M_p[c]	$Đ$[d]
1	$\mathbf{P_{H(10k)}}$	11200	12000	12000	1.07
2	$\mathbf{P_{H(25k)}}$	25000	29200	25900	1.16
3	$\mathbf{P_{H(27k)}}$	27100	30600	28600	1.13
4	$\mathbf{P_{V(10k)}}$	13700	14500	14600	1.06
5	$\mathbf{P_{V(25k)}}$	25000	28100	26200	1.15
6	$\mathbf{P_{VS\text{-}7}}$	11000	23700	15400	2.16

[a]Number average molecular weight, determined by SEC with RI detector. [b]Weight average molecular weight, determined by SEC with RI detector. [c]Peak molecular weight, determined by SEC with RI detector. [d]Dispersity (= M_w/M_n), determined by SEC.

Supplementary Table 2. Formation of silicone elastomers.

Entry	$\mathbf{P_H}$	$\mathbf{P_V}$	$\mathbf{S_E}$	G'_e /kPa[a]
1	$\mathbf{P_{H(10k)}}$	$\mathbf{P_{V(10k)}}$	$\mathbf{S_{E(10k,10k)}}$	28
2	$\mathbf{P_{H(10k)}}$	$\mathbf{P_{VS\text{-}7}}$	$\mathbf{S_{E(10k,\ VS\text{-}7)}}$	120
3	$\mathbf{P_{H(25k)}}$	$\mathbf{P_{V(25k)}}$	$\mathbf{S_{E(25k,25k)}}$	230
4	$\mathbf{P_{H(27k)}}$	$\mathbf{P_{V(10k)}}$	$\mathbf{S_{E(27k,10k)}}$	27
5	$\mathbf{P_{H(27k)}}$	$\mathbf{P_{VS\text{-}7}}$	$\mathbf{S_{E(27k,VS\text{-}7)}}$	33

[a]The equilibrated G' of $\mathbf{S_E}$ reached after at least two hours after curing a mixture of $\mathbf{P_H}$ and $\mathbf{P_V}$.
