# Supplementary material for: Synergetic binary organocatalyzed ring opening polymerization for the precision synthesis of polysiloxanes
Source: Commun Chem. 2024 Mar 21;7:61. doi: 10.1038/s42004-024-01140-3 (PMC10957864; doi:10.1038/s42004-024-01140-3)
Supplement: Supplementary file 3 — Description of Additional Supplementary Files [file 42004_2024_1140_MOESM3_ESM.pdf]

Description of Additional Supplementary Files

File name: Supplementary Data 1

Description: Numerical data for plots of conversion and Đ against time

File name: Supplementary Data 2

Description: 1H NMR spectra
