# Supplementary material for: Synergetic binary organocatalyzed ring opening polymerization for the precision synthesis of polysiloxanes
Source: Commun Chem. 2024 Mar 21;7:61. doi: 10.1038/s42004-024-01140-3 (PMC10957864; doi:10.1038/s42004-024-01140-3)
Supplement: Supplementary file 5 — Supplementary Data 2 [file 42004_2024_1140_MOESM5_ESM.pdf]

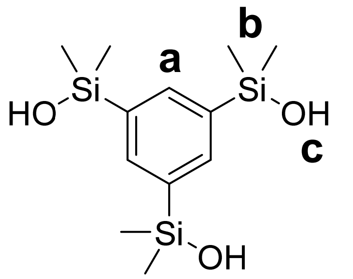

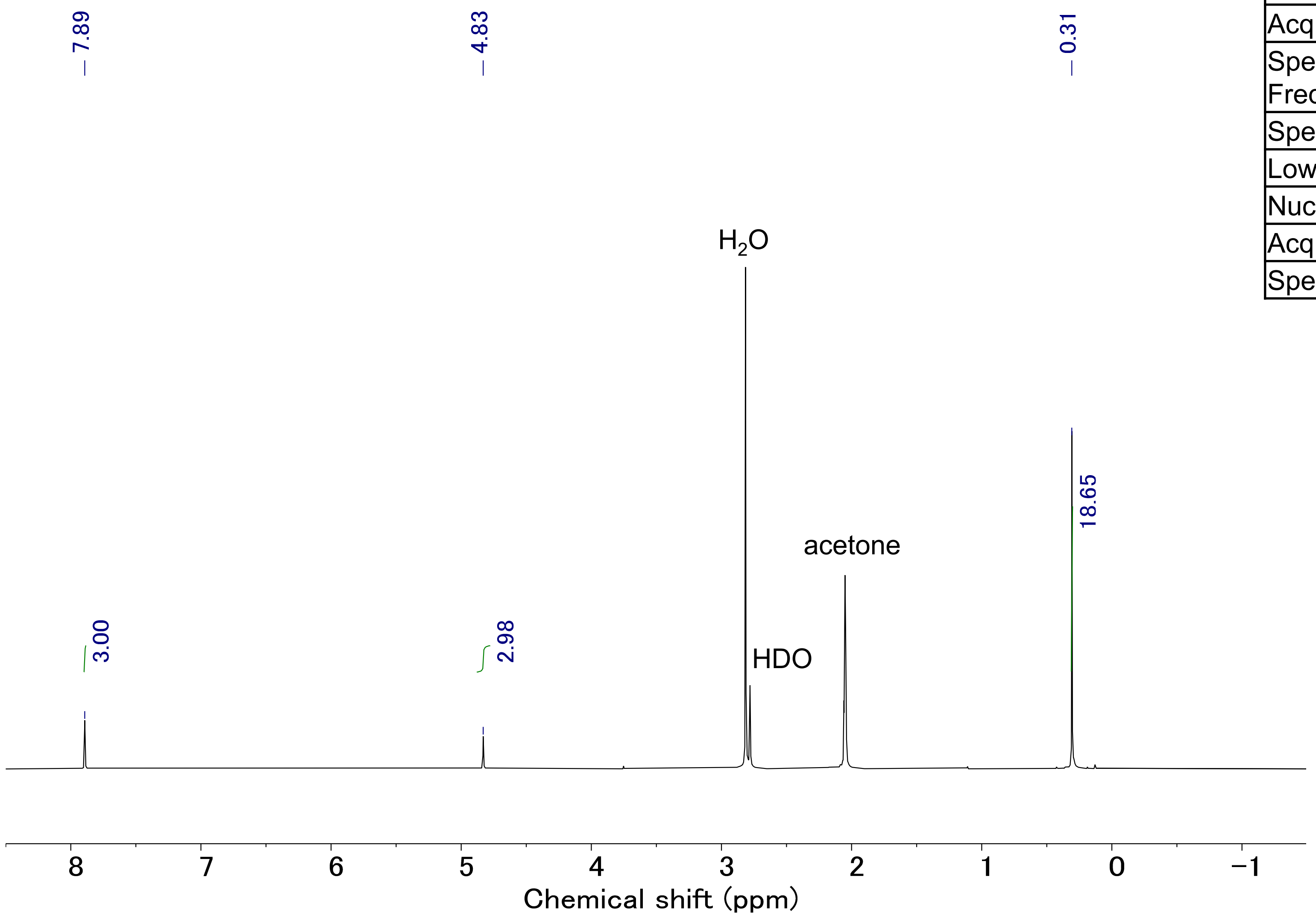

Solvent	Acetone
Temperature	299.4
Pulse Sequence	zg30
Experiment	1D
Number of Scans	16
Receiver Gain	203.0
Relaxation Delay	1.0000
Pulse Width	12.0000
Acquisition Time	3.2768
Spectrometer Frequency	500.13
Spectral Width	10000.0
Lowest Frequency	-1919.6
Nucleus	1H
Acquired Size	32768
Spectral Size	65536

1

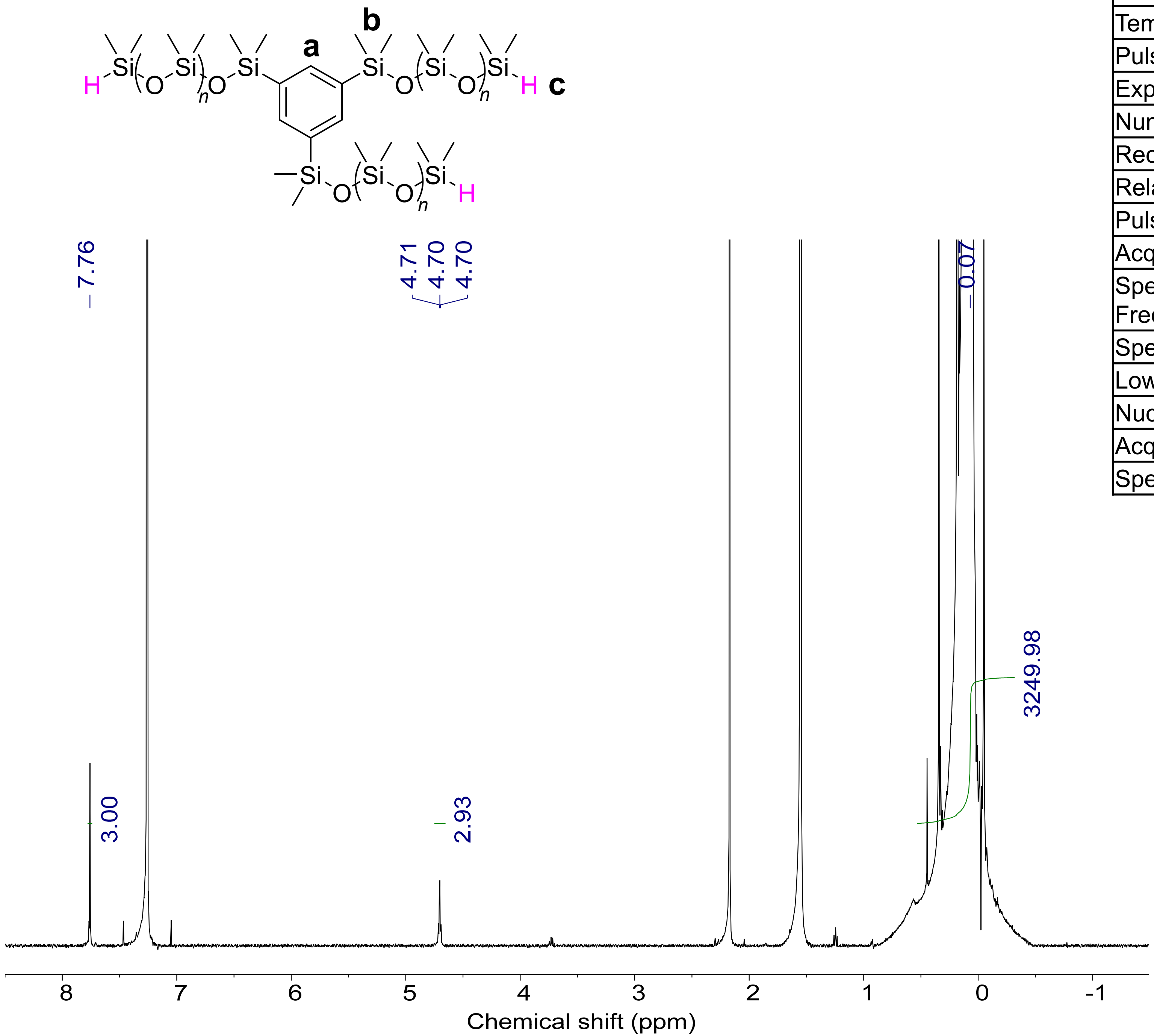

Solvent	CDCl3
Temperature	298.5
Pulse Sequence	zg30
Experiment	1D
Number of Scans	16
Receiver Gain	79.5
Relaxation Delay	1.0000
Pulse Width	12.0000
Acquisition Time	3.2768
Spectrometer Frequency	500.03
Spectral Width	10000.0
Lowest Frequency	-1925.2
Nucleus	1H
Acquired Size	32768
Spectral Size	65536

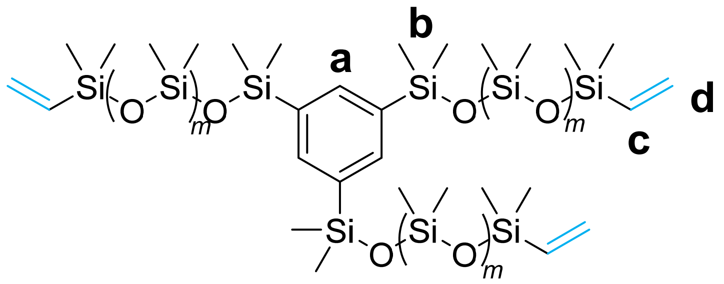

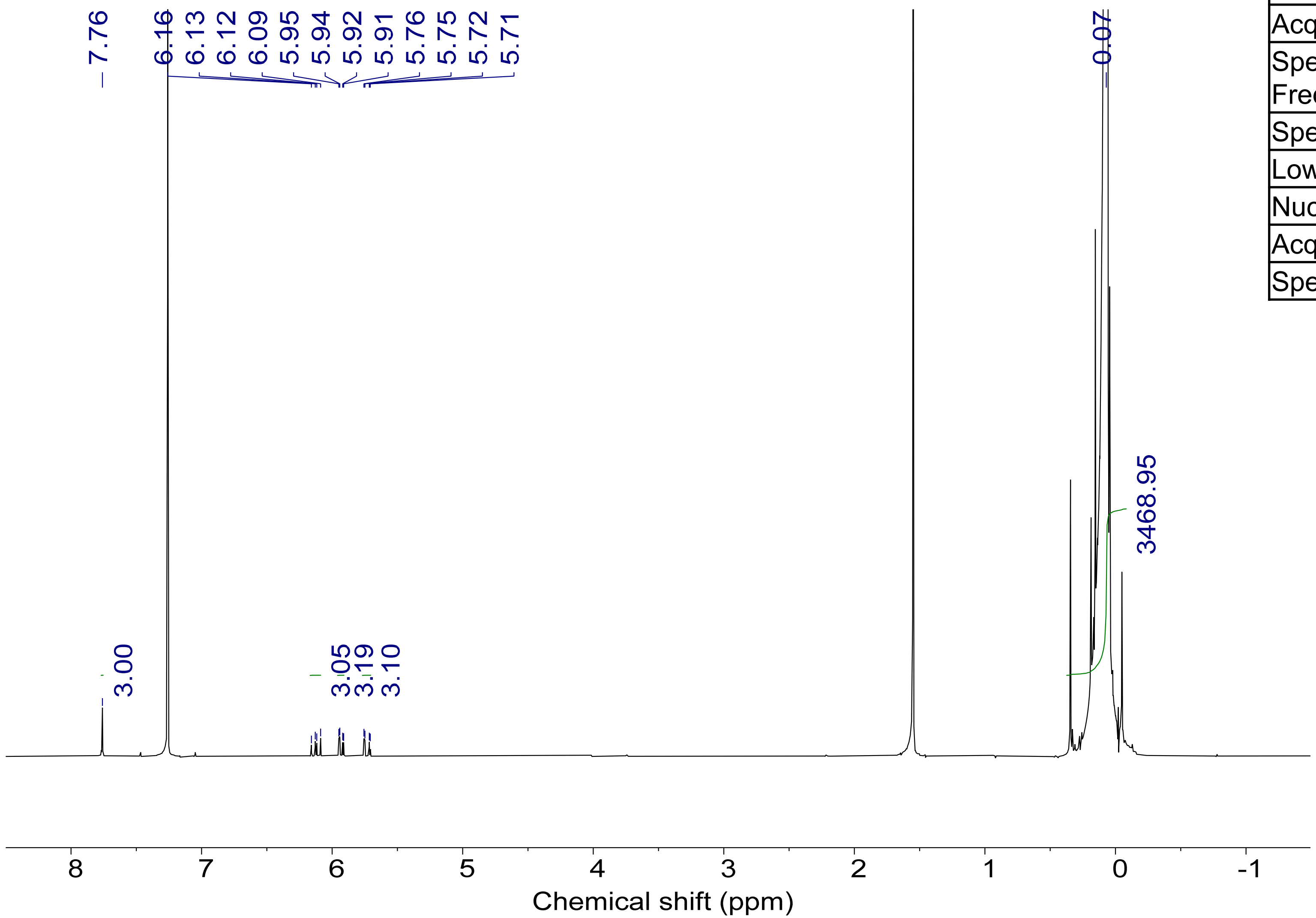

Solvent	CDCl3
Temperature	298.5
Pulse Sequence	zg30
Experiment	1D
Number of Scans	16
Receiver Gain	79.5
Relaxation Delay	1.0000
Pulse Width	12.0000
Acquisition Time	3.2768
Spectrometer Frequency	500.03
Spectral Width	10000.0
Lowest Frequency	-1925.2
Nucleus	1H
Acquired Size	32768
Spectral Size	65536
